# An analysis of socioeconomic factors on multiple chronic conditions and its economic burden: evidence from the National Health Service Survey in Yunnan Province, China

**DOI:** 10.3389/fpubh.2023.1114969

**Published:** 2023-05-03

**Authors:** Puxian Peng, Jing Li, Liping Wang, Zhonghua Ai, Churou Tang, Songyuan Tang

**Affiliations:** ^1^Institute of Health Studies, School of Public Health, Kunming Medical University, Kunming, Yunnan, China; ^2^Yunnan Health Development Research Center, Kunming, China; ^3^Department of Biology, University of Rochester, Rochester, NY, United States

**Keywords:** chronic diseases, multimorbidity, economic burden, socio-economic factors, Chinese

## Abstract

**Background:**

The economic burden of multiple chronic conditions (MCCs) and its socio-economic influencing factors have widely raised public concerns. However, there are few large population-based studies on these problems in China. Our study aims at determining the economic burden of MCCs and associated factors specific to multimorbidity among middle-aged and older individuals.

**Methods:**

As our study population, we extracted all 11,304 participants over 35 years old from the 2018 National Health Service Survey (NHSS) in Yunnan. Economic burden and socio-demographic characteristics were analyzed with descriptive statistics. Chi-square test and generalized estimating equations (GEE) regression models were used to identify influencing factors.

**Results:**

The prevalence of chronic diseases was 35.93% in 11,304 participants and the prevalence of MCCs increased with age, was 10.12%. Residents who lived in rural areas were more likely to report MCCs than those who lived in urban areas (adjusted *OR* = 1.347, 97.5% *CI*: 1.116–1.626). Ethnic minority groups were less likely to report MCCs than those of Han (*OR* = 0.752, 97.5% *CI*: 0.601–0.942). Overweight or obese people were more likely to report MCCs than people with normal weight (*OR* = 1.317, 97.5% *CI*: 1.099–1.579). The *per capita* expenses of 2 weeks’ illness, *per capita* hospitalization expenses, annual household income, annual household expenses, and annual household medical expenses of MCCs were ¥292.90 (±1427.80), ¥4804.22 (±11851.63), ¥51064.77 (±52158.76), ¥41933.50 (±39940.02) and ¥11724.94 (±11642.74), respectively. The *per capita* expenses of 2 weeks’ illness, *per capita* hospitalization expenses, annual household income, annual household cost, and annual household medical expenses of hypertensive co-diabetic patients were more compared to those with other three comorbidity modes.

**Conclusion:**

The prevalence of MCCs was relatively high among middle-aged and older individuals in Yunnan, China, which bought a heavy economic burden. This encourages policy makers and health providers to pay more attention to the behavioral/lifestyle factors, that contribute to multimorbidity to a great extent. Furthermore, health promotion and education in terms of MCCs need to be prioritized in Yunnan.

## Introduction

Chronic disease conditions are important public health problems in both developed and developing countries, which seriously threaten people’s lives and health, bringing a heavy economic burden. In western countries, among the whole population, the prevalence of multiple diseases ranges from 20 to 30%, while the prevalence increased to the range from 55 to 98% in the older individuals (1). At present, MCCs have become the primary risk factors for health hazards, accounting for more than 80% of the 10.3 million annual deaths in China, accounting for 68.6% of the total economic burden (2).

Multiple chronic conditions (MCCs) is the focus of chronic disease conditions. Most recent studies define MCCs as two or more chronic diseases in a person at the same time (3). The impact of MCCs on people’s health and life is increasingly significant, along with serious economic burden. The economic burden of MCCs and its socio-economic influencing factors have widely raised public concerns worldwide. According to a systematic review conducted in 2011, about one-third of adults in the world suffer from MCCs (4, 5). The prevalence of MCCs in low-income and middle-income countries has recently been estimated at 10–11%, and it is expected to increase in the next few years (6). Due to the complexity of symptoms and high mortality, the treatment requirements of MCCs are more complicated than those of a single chronic disease, resulting in patients with MCCs often cannot get cost-effective treatment (7). In most countries, it is challenging to meet the care needs of patients with chronic diseases (8). This situation may increase the financial and medical burden of these patients, given the decline in quality of life, and cause greater damage to their physical and mental health (7, 9).

Some previous studies on MCCs which only considered socio-demographic factors and the overall economic burden of the MCCs, with few large population-based studies detailing the socio-economic determinants and economic burden of specific combinations of MCCs. Studies have revealed that patients with MCCs have more prescriptions and incur more medical expenses than people with one or no chronic condition (10, 11). Meanwhile, many policy studies on the prevention and management of chronic diseases, are only limited to studying chronic diseases individually (12–15). There is no policy study on chronic disease co-morbidities. Therefore, as the burden of MCCs increases, there is an urgent need to investigate MCCs within a larger population and generalize more effective management methods of MCCs at all contact points, as well as to deepen the understanding of multiple socio-economic factors affecting the combination of chronic and co-morbid diseases and the economic burden they impose. Consequently, a theoretical basis will be provided to policymakers on developing chronic disease control program.

Our study population are restricted to the Yunnan province. Yunnan, located on the plateau mountains of southwest China, is one of the poorest areas in China. The area has unique geographical characteristics, different distribution characteristics of ethnic minorities and tobacco epidemic. There is still a lack of research on MCCs in Yunnan. The NHSS is a national survey of the entire Chinese population. The purpose of NHSS is to objectively reflect the achievements and problems of health reform and development, and predict the changing trends of health service needs, demands and utilization by understanding the residents’ health service needs, demands and utilization as well as their satisfaction with medical services, so as to provide a basis for evaluating the implementation effect of health reform and promoting the healthy action. Thus, based on the data from NHSS, this study made a preliminary study on multiple chronic diseases in Yunnan province.

In the present study, we aim to determine the economic burden of MCCs and associated factors specific to multimorbidity among middle-aged and older individuals. Firstly, we describe the distribution of MCCs among middle-aged and older individuals in Yunnan. Then estimates the relationship between demographic characteristics, socio-economic factors and MCCs by using the generalized estimating equations (GEE) model. Finally, we compare the economic burden of various comorbidities. Our research results can serve as a theoretical basis for health policy makers and implementers, and as references for other related studies of MCCs.

## Materials and methods

### Data source

**Figure 1:**
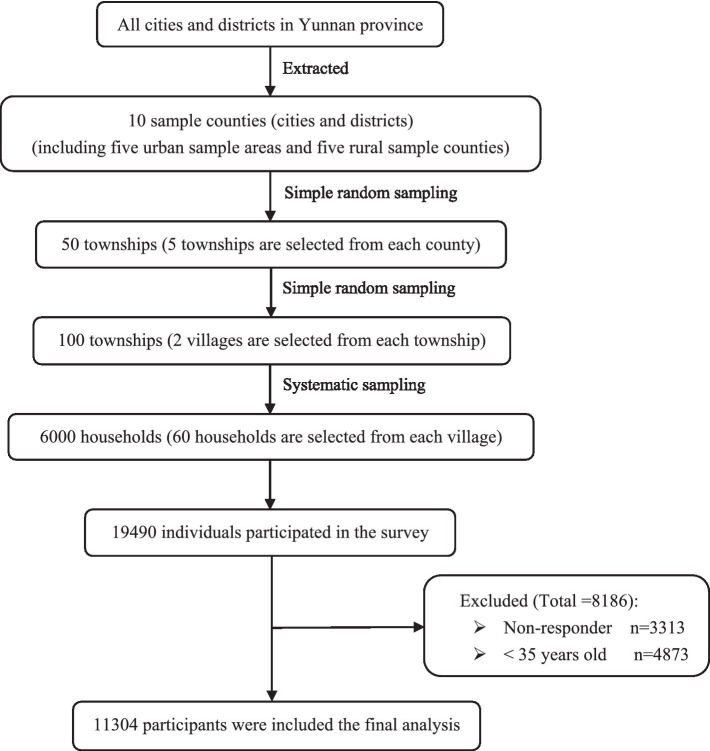
Flowchart of the selected participants from the NHSS.

The primary data used in this study were derived from the Sixth (2018) National Health Service Survey (NHSS) in Yunnan province. According to the unified requirements and deployment of the National Health and Wellness Commission, the survey samples followed the principle of economic and effective sampling, and adopted the method of multistage stratified cluster random sampling. A total of 10 sample counties (cities and districts) were selected: five urban sample areas and five rural sample counties. Five townships are selected from each county (city, district), and the quota (economy, region) is used for simple random sampling. Two villages (neighborhood committees) are selected from each township, and the quota (economy, region) is used for simple random sampling. Each village (neighborhood committee) selects 60 households and adopts systematic sampling. A total of 50 townships and 100 villages (neighborhood committees) were selected in the province, including 6,000 households (actually 6,010 households) with 19,490 individuals. Data is gathered by trained and qualified investigators, using the unified mobile terminal to carry out the household inquiry investigation; quality control is carried out in all aspects of the investigation. The survey results show that the answer rate of people over 15 is 83.0%. Our research sample comprises 11,304 respondents aged 35 and above from this health service survey (Figure 1).

### Study variables selection

#### Dependent variable

The dependent variable is multiple chronic conditions. It is determined by the respondents’ current chronic disease status. The dependent variable is classified as no chronic condition, one chronic condition, and two or more chronic conditions.

#### Independent variable

Independent variables were categorized into three major groups: demographic variables (such as age, gender, ethnicity, marital status, area of resident), social and economic status, and related health variables. Demographic variables are included in our analysis to reduce the impact of population differences. Social and economic status includes income level, education level and employment status. Related health variables include smoking status, drinking status, health status, physical examination, physical exercise and BMI. Self-reported health status was assessed by using Visual Analog Scale (VAS) in the questionnaire, and it was divided into 5 grades (16, 17) as in previous studies. See Table 1 for a detailed description of dependent and independent variables.

**Table 1:** Sociodemographic prevalence of chronic condition among respondents in Yunnan province in 2018 [*n* (%)].

Variable	No chronic condition	One chronic condition	Two or more chronic conditions	*χ* ^2^	*p*-value
Overall (*N* = 11,304)	7,243 (64.07)	2,917 (25.81)	1,144 (10.12)	–	–
Area of residence				23.061	<0.001
Urban area	3,688 (66.21)	1,368 (24.56)	514 (9.23)		
Rural area	3,555 (62.00)	1,549 (27.01)	630 (10.99)		
Age group				1034.4	<0.001
35–44	2,291 (82.47)	416 (14.97)	71 (2.56)		
45–54	2,393 (69.55)	828 (24.06)	220 (6.39)		
55–64	1,338 (55.56)	733 (30.44)	337 (14.00)		
65+	1,221 (45.61)	940 (35.11)	516 (19.28)		
Sex				25.297	<0.001
Male	3,706 (66.37)	1,353 (24.23)	525 (9.40)		
Female	3,537 (61.84)	1,564 (27.34)	619 (10.82)		
Ethnicity				57.024	<0.001
Han	5,237 (62.30)	2,234 (26.57)	936 (11.13)		
Others	2006 (69.24)	683 (23.58)	208 (7.18)		
Marital status				74.158	<0.001
Single	188 (73.15)	60 (23.35)	9 (3.50)		
Married	6,246 (65.14)	2,417 (25.21)	925 (9.65)		
Divorce/widowed/others	809 (55.45)	440 (30.16)	210 (14.39)		
Education				73.100	<0.001
Primary and below	4,611 (61.60)	2077 (27.75)	797 (10.65)		
Secondary	1,566 (69.82)	474 (21.13)	203 (9.05)		
Pre-U/Junior College/Diploma	514 (63.85)	214 (26.58)	77 (9.57)		
University and above	552 (71.60)	152 (19.71)	67 (8.69)		
Employment				680.980	<0.001
Employed	5,233 (72.04)	1,559 (21.46)	472 (6.50)		
Retired	369 (41.41)	312 (35.02)	210 (23.57)		
Unemployment	1,641 (52.11)	1,046 (33.22)	462 (14.67)		
Household income				15.655	0.0476
<10,000	1931 (63.15)	841 (27.50)	286 (9.35)		
10,000–30,000	2,672 (65.31)	1,023 (25.01)	396 (9.68)		
30,000–50,000	1,113 (62.77)	467 (26.34)	193 (10.89)		
50,000–100,000	1,128 (63.65)	438 (24.72)	206 (11.63)		
>100,000	399 (65.41)	148 (24.26)	63 (10.33)		
Physical exercise (no. times per week)				145.468	<0.001
0	5,015 (66.53)	1890 (25.07)	633 (8.40)		
1–2	364 (69.33)	119 (22.67)	42 (8.00)		
3–5	410 (63.27)	172 (26.54)	66 (10.19)		
≥6	1,454 (56.07)	736 (28.38)	403 (15.54)		
Smoking				44.601	<0.001
Yes	2,834 (67.88)	987 (23.64)	354 (8.48)		
No	4,409 (61.85)	1930 (27.07)	790 (11.08)		
Drinking				156.590	<0.001
Yes	2,629 (72.13)	760 (20.85)	256 (7.02)		
No	4,614 (60.25)	2,157 (28.16)	888 (11.59)		
State of health				1023.900	<0.001
Poor (0–40)	331 (38.71)	335 (39.18)	189 (22.11)		
Medium (41–60)	1,566 (50.53)	1,005 (32.43)	528 (17.04)		
Good (61–80)	3,120 (67.24)	1,167 (25.15)	353 (7.61)		
Better (81–100)	2,205 (82.89)	391 (14.70)	64 (2.41)		
Physical examination				135.740	<0.001
Yes	2,715 (58.08)	1,359 (29.08)	600 (12.84)		
No	4,528 (68.29)	1,558 (23.50)	544 (8.21)		
BMI (kg/m^2^)				105.140	<0.001
Underweight	865 (61.57)	393 (27.97)	147 (10.46)		
Normal	4,409 (67.76)	1,543 (23.72)	554 (8.52)		
Overweight/obese	1949 (58.08)	969 (28.87)	438 (13.05)		

### Statistical analysis

Economic burden and socio-demographic characteristics were analyzed with descriptive statistics. The Chi-square test was used to explore the associated factors of MCCs. Associations among MCCs and factors were estimated using GEE models which further controlled for possible influencing factors of the internal connection among family members with households as the sampling unit. The GEE model assumes that the probability of parameters is binomial distribution with a logit link function. Therefore, due to the nature of the complex data structure of NHSS, the GEE model was used to adjust the relevant personal responses (18). At the recommendation of Liang and Zeger (19), we are interested in OR values in this study, which should be analyzed using GEE2. The SPSS software (Version 26) was used to carry out all statistical analyses. The significance level was set at less than 0.05, two-tailed, except for GEE. Considering that the dependent variables in GEE involve multiple comparisons, we adjusted the significance level according to Bonferroni method, and the adjusted significance level is 0.05/2 = 0.025.

The calculation formula of GEE2 model is as follows:


$$\sum_{i=1}^{K}U_{i}\left[\beta ,\hat{\alpha }\left\{\beta ,\hat{\phi }\left(\beta \right)\right\}\right]=0$$

β : Coevariate coefficient

ϕ : Dispersion parameter

α : Related parameters

Both ϕ and α are functions of β. Only given the estimated values of ϕ and α can the solution of β be obtained.

## Results

### Characteristics of study population with MCCs

Table 1 presents the major characteristics of all 11,304 respondents aged 35 years old and over. The prevalence of chronic diseases was 35.93% and the prevalence of MCCs was 10.12%, increased with age. In urban areas, the prevalence of one chronic condition in participants was 24.56, and 9.23% of the study population suffered from two or more chronic diseases. The prevalence rates of one chronic disease and two or more chronic diseases were 27.01 and 10.99% in rural areas, respectively. Among the age groups, the highest prevalence rates of multiple chronic diseases were 14.00% (55–64 years old) and 19.28% (65 years old or over). As age increased, the prevalence of one chronic disease and two or more chronic diseases significantly increased (*p* < 0.001). The prevalence of one chronic disease were 24.23% (male) and 27.34% (female), respectively, and the comorbidity rate of chronic diseases in females (10.82%) is higher than that in males (9.40%) (*p* < 0.001). The prevalence rates of one chronic disease in Han and other minority groups were 26.57 and 23.58%, respectively, and the comorbidity rates of chronic diseases were 11.13 and 7.18%, respectively. The remaining characteristics of study population with MCCs are shown in Table 1.

### Gender-specific chronic diseases

The top 8 chronic disease prevalence rates among males from high to low were hypertension (14.89%), intervertebral disc disease (3.84%), diabetes (3.81%), acute and chronic gastroenteritis (2.35%), cerebrovascular diseases (2.29%), rheumatoid arthritis (1.77%), other chronic obstructive pulmonary diseases (1.65%), other sports diseases (1.61%) respectively. The top 10 chronic disease prevalence rates among females from high to low were hypertension (18.30%), intervertebral disc disease (5.35%), diabetes (4.58%), rheumatoid arthritis (3.02%), acute and chronic gastroenteritis (2.94%), other sports diseases (1.64%), cerebrovascular diseases (1.59%), other types of heart disease (1.15%), cholelithiasis and cholecystitis (1.12), other chronic obstructive pulmonary diseases (1.03%) respectively. The prevalence of other chronic diseases in males and females were less than 1% (see Table 2).

**Table 2:** Prevalence of chronic diseases among respondents in Yunnan province in 2018 [*n* (%)].

Cis-position	Male		Female	
	Types of chronic diseases	Number of patients	Types of chronic diseases	Number of patients
1	Hypertension	832 (14.89)	Hypertension	1,047 (18.30)
2	Intervertebral disc disease	214 (3.83)	Intervertebral disc disease	306 (5.35)
3	Diabetes	213 (3.81)	Diabetes	262 (4.58)
4	Acute and chronic gastroenteritis	131 (2.35)	Rheumatoid arthritis	173 (3.02)
5	Cerebrovascular diseases	128 (2.29)	Acute and chronic gastroenteritis	168 (2.94)
6	Rheumatoid arthritis	99 (1.77)	Other sports diseases	94 (1.64)
7	Other chronic obstructive pulmonary diseases	92 (1.65)	Cerebrovascular diseases	91 (1.59)
8	Other sports diseases	90 (1.61)	Other types of heart disease	66 (1.15)
9	Urinary calculus	54 (0.97)	Cholelithiasis and cholecystitis	64 (1.12)
10	Prostate hyperplasia or inflammation	49 (0.88)	Other chronic obstructive pulmonary diseases	59 (1.03)
11	Other types of heart disease	46 (0.82)	Other neurological diseases	38 (0.66)
12	Other respiratory diseases	37 (0.66)	Other digestive diseases	37 (0.65)

### Result of multivariate analysis of MCCs

Table 3 indicates the results of GEE models by taking MCCs occurrence as the dependent variable. First, we screened the noticeable demographic covariates of MCCs at a lower significance level of 0.025; all demographic covariates, such as area of residence, age group, sex, marital status, and education were included in the classified variables analysis. Second, GEE models 1 showed the adjusted associations between no chronic condition versus two or more chronic conditions and demographic covariates. Finally, in model 2, we further incorporated the product term of two or more chronic vs. one chronic condition and demographic covariates.

**Table 3:** Results of generalized estimating equations of multiple chronic conditions in Yunnan province in 2018.

Variables	Two or more chronic conditions vs. no chronic condition		Two or more chronic conditions vs. one chronic condition	
	*OR*	97.5% *CI*	*OR*	97.5% *CI*
*Area of residence (ref: Urban area)*				
Rural area	1.563	**1.290–1.895**	1.347	**1.116–1.626**
*Age group (ref: 35–44)*				
45–54	2.556	**1.822–3.587**	1.392	0.980–1.977
55–64	5.896	**4.205–8.266**	2.231	**1.584–3.144**
65+	7.709	**5.438–10.929**	2.540	**1.786–3.612**
*Sex (ref: male)*				
Female	0.776	**0.615–0.979**	0.903	0.709–1.149
*Ethnicity (ref: Han)*				
Others	0.593	**0.471–0.747**	0.752	**0.601–0.942**
*Marital status (ref: single)*				
Married	1.863	0.819–4.240	1.863	0.794–4.367
Divorce/widowed/others	1.568	0.671–3.667	1.880	0.782–4.521
*Education (ref: primary and below)*				
Secondary	1.149	0.910–1.450	1.274	**1.010–1.607**
Pre-U/Junior College/diploma	1.196	0.825–1.735	0.959	0.666–1.379
University and above	1.507	0.995–2.283	1.498	0.978–2.293
*Household income (ref: <10,000)*				
10,000–30,000	1.181	0.942–1.482	1.235	0.992–1.539
30,000–50,000	1.352	**1.014–1.803**	1.307	0.994–1.719
50,000–100,000	1.380	**1.030–1.851**	1.358	**1.024–1.802**
>100,000	1.394	0.872–2.226	1.371	0.866–2.170
*Physical exercise (no. times per week) (ref: 0)*				
1–2	1.239	0.792–1.937	1.260	0.809–1.962
3–5	1.371	0.958–1.962	1.123	0.783–1.612
≥6	2.103	**1.691–2.617**	1.571	**1.275–1.935**
*Smoking (ref: yes)*				
No	1.043	0.819–1.329	1.047	0.811–1.351
*Drinking (ref: yes)*				
No	1.834	**1.474–2.280**	1.150	0.914–1.447
*State of health [ref: poor (0–40)]*				
Medium (41–60)	0.543	**0.418–0.706**	0.795	0.620–1.019
Good (61–80)	0.167	**0.125–0.223**	0.432	**0.331–0.563**
Better (81–100)	0.046	**0.030–0.068**	0.225	**0.152–0.333**
*Physical examination (ref: yes)*				
No	0.720	**0.606–0.855**	0.939	0.792–1.113
*BMI (kg/m^2^) (ref: normal)*				
Underweight	0.722	**0.557–0.936**	0.907	0.700–1.174
Overweight or obese	2.282	**1.900–2.741**	1.317	**1.099–1.579**

The bold values indicates that *p*-value is less than 0.025, which has significant statistical significance.

From the results of model 2: who residents lived in rural areas were more likely to report MCCs than those who lived in urban areas (adjusted *OR* = 1.347, 97.5% *CI*: 1.116–1.626); compared to people 35–44 years old, those with 55–64 and 65 years old and above were more likely to report MCCs (*OR* = 2.231, 97.5% *CI*: 1.584–3.144; *OR* = 2.540, 97.5% *CI*: 1.786–3.612, respectively). People of other minorities were less likely to suffer from MCCs than Han (*OR* = 0.752, 97.5% *CI*: 0.601–0.942). Compared with those with primary school education and below, people with junior high school education were more likely to suffer from MCCs (*OR* = 1.274, 97.5% *CI*: 1.010–1.607). People with an annual household income of ¥100,000 were more likely to report MCCs than those less than ¥10,000 (*OR* = 1.358, 97.5% *CI*: 1.024–1.802). People who exercise five or more times a week were more likely to report MCCs than those who did not exercise (*OR* = 1.571, 97.5% *CI*: 1.275–1.935); those who were in good and better health were less likely to suffer from MCCs than those in poor health (*OR* = 0.432, 97.5% *CI*: 0.331–0.563; *OR* = 0.225, 97.5% *CI*: 0.152–0.333, respectively). Overweight or obese people were more likely to report MCCs than normal shape people (*OR* = 1.317, 97.5% *CI*: 1.099–1.579).

### Multimorbidity of hypertension and diabetes

Table 4 presents a total of 243 participants who reported hypertension and diabetes, accounting for 21.24% of 1,144 patients with MCCs. There were 503 (43.97%) respondents with hypertension and non-diabetic chronic diseases. The number of patients with diabetes and non-hypertensive chronic diseases and non-hypertensive and non-diabetic chronic diseases were 49 (4.28%) and 349 (30.51%), respectively. In urban area, there were 149 (13.02%) participants with hypertension and diabetes. There were 94 (8.22%) participants with hypertension and diabetes in rural area. The number of patients with hypertension and diabetes were 99 (8.65%) in males and 144 (12.59%) in females. Among the age groups, the largest number of patients with hypertension and diabetes was 77 (6.73%) (55–64 years old) and 131 (11.45%) (65 years old or over). The percentage of hypertension complicated with diabetes in the Han minority and others were 17.05 and 4.20%, respectively.

**Table 4:** Demographic characteristics, multimorbidity of hypertension and diabetes in Yunnan province in 2018 [*n* (%)].

Characteristics	Hypertension and Diabetes	Hypertension and non-diabetic chronic diseases	Diabetes and non-hypertensive chronic diseases	Non-hypertensive and non-diabetic chronic diseases
Overall	243 (21.24)	503 (43.97)	49 (4.28)	349 (30.51)
*Area of residence*				
Urban area	149 (13.02)	221 (19.32)	22 (1.92)	122 (10.66)
Rural area	94 (8.22)	282 (24.65)	27 (2.36)	227 (19.84)
*Sex*				
Male	99 (8.65)	255 (22.29)	21 (1.84)	150 (13.11)
Female	144 (12.59)	248 (21.68)	28 (2.45)	199 (17.40)
*Age group*				
35–44	1 (<0.01)	19 (1.66)	1 (<0.01)	50 (4.37)
45–54	34 (2.97)	84 (7.34)	12 (1.05)	90 (7.87)
55–64	77 (6.73)	143 (12.5)	15 (1.31)	102 (8.92)
65+	131 (11.45)	257 (22.47)	21 (1.84)	107 (9.35)
*Ethnicity*				
Han	195 (17.05)	400 (34.97)	44 (3.85)	297 (25.96)
Others	48 (4.20)	103 (9.00)	5 (0.44)	52 (4.55)

### Socio-economic burden of hypertension and diabetes

As shown in Table 5, a total of *per capita* expenses of 2 weeks’ illness of patients with MCCs was ¥216.05 (±1067.50) while *per capita* hospitalization expenses, annual household income, annual household expenditure, annual household medical expenses of participants were ¥4163.34 (±11518.77), ¥38918.28 (±40613.14), ¥35624.96 (±36720.35) and ¥10647.42 (±12556.92), respectively. The *per capita* expense of 2 weeks’ illness of hypertensive co-diabetic patients was ¥292.90 (±1427.80), while *per capita* hospitalization expenses, annual household income, annual household expenditure, and annual household medical expenses of participants were ¥4804.22 (±11851.63), ¥51064.77 (±52158.76), ¥41933.50 (±39940.02) and ¥11724.94 (±11642.74), respectively. The *per capita* expenses of 2 weeks’ illness, *per capita* hospitalization expenses, annual household income, annual household expenditure, and annual household medical expenses of hypertensive co-diabetic patients were more compared to the patients with the other three comorbidity modes (see Table 5).

**Table 5:** Mean value of economic index measurements by socio-economic variables among patients with MCCs in Yunnan province in 2018 [mean (SD), ¥].

Socio-economic variables	Overall	Hypertension and diabetes	Hypertension and nondiabetic chronic diseases	Diabetes and non-hypertensive chronic disease	Non-hypertensive and nondiabetic chronic disease
*Per capita* expenses of 2 weeks’ illness	216.05 (1067.50)	292.90 (1427.80)	226.28 (1140.96)	137.33 (428.64)	158.28 (643.04)
*Per capita* hospitalization expenses	4163.34 (11518.77)	4804.22 (11851.63)	3918.69 (12121.89)	3869.16 (7595.06)	4111.03 (10853.78)
Annual household income	38918.28 (40613.14)	51064.77 (52158.76)	35685.29 (36518.18)	30622.45 (26174.20)	36285.32 (37049.39)
Annual household expenditure	35624.96 (36720.35)	41933.50 (39940.02)	34115.57 (38477.39)	30551.02 (20846.72)	34120.29 (32967.20)
Annual household medical expenses	10647.42 (12556.92)	11724.94 (11642.74)	10367.97 (13103.69)	11063.24 (12623.31)	10241.55 (12362.72)

## Discussion

### Summary of main findings and comparison with existing literature

In the current study, we analyzed the aggregated cross-sectional data from NHSS in Yunnan Province in 2018 to determine the economic burden of MCCs and associated factors specific to multimorbidity among middle-aged and older individuals. The prevalence of chronic diseases was 35.93% in 11,304 participants; the prevalence of MCCs was 10.12%. The prevalence of MCCs increased with age, and this finding has been consistently reported in previous studies (20). A reasonable explanation for the increasing prevalence of MCCs with age is that it may be associated with many age-related changes in the individual’s physiological state, such as changes in metabolism, immune response, and organ function (21). Compared to other Chinese studies, the prevalence of chronic diseases is higher than 30.3% while the prevalence of MCCs is lower than 12.3% reported by Wong’s team (22), based on a sample of respondents over 18 years old. The difference in estimated values among different studies was caused primarily by researchers’ choice of research group/population, the number of chronic diseases considered in the study, or both (23). For example, some studies defined MCCs differently as three or more chronic diseases.

This study evaluated the effects of eight socioeconomic factors (area of residence, age, ethnicity, educational level, household income, physical exercise, state of health and body mass index) on MCCs in model 2 of Table 3. Physical state of health is a positive factor for MCCs. The prevalence of MCCs in rural residents is higher than that in urban residents. People living in rural areas may have less access to health care and other services than those in urban areas (24). While compared with urban residents, people living in rural areas are often poorer and less educated (25, 26). People of other minorities were less likely to experience MCCs than Han, which may be caused by the association between ethnicity and BMI. Previous studies in China have found that the risk of obesity and central obesity among ethnic minorities is reduced (27, 28), which result from genetic and environmental heterogeneity (29). Compared to those with a primary school education or below, those with a junior high school education had a much higher risk of experiencing MCCs. The reason behind this inconsistency (29) with other studies needs to be further studied. Respondents with an annual household income of ¥50,000–100,000 had much higher odds of experiencing MCCs compared to those less than ¥10,000. However, behavioral interpretation is not possible because the link between income and education is significant even after controlling for various healthy behaviors.

Surprisingly, individuals who exercised ≥6 times per week had a higher risk to occur MCCs than those who did not exercise. This finding is consistent with recent studies in high-income, middle-income and low-income countries, which reported that higher levels of physical activity were associated with the prevalence of MCCs (30, 31). We propose that individuals with MCCs started to exercise more because of preexisting illnesses. Overweight and obese individuals are more likely to have MCCs than those with normal weight. This result is consistent with other studies around the world (30, 32). Studies have shown that both underweight and normal body weight can help reduce the incidence of chronic diseases (33, 34). Therefore, the inclusion of body mass index to identify and monitor obesity should be a health care priority, along with weight management and targeted control of other risk factors such as MCCs (35). The current perspective is that any movement from unhealthy to healthy behaviors has significant health benefits (36).

The results of model 1 in Table 3 demonstrated that, in addition to the above socio-economic factors, sex and alcohol consumption also affected the development of MCCs. People who did not drink alcohol were more likely to develop MCCs than those who drank alcohol. This is most likely because patients are forced to abstain from alcohol after learning of their illness. Meanwhile, female had a lower prevalence of MCCs than male. This finding contradicts the previous research (37–39), which showed that MCCs tend to be more common in female than male. However, sex was not independently associated with MCCs. Men were more likely to smoke and drink alcohol than women (40), which may explain why men are more prone to experience MCCs. These findings can provide added value in designing guidelines for different chronic conditions that can be assessed in groups or individually. These predictions can be useful for frontline medical staff seeking the best approach to care for multi-disease patients.

Table 5 indicates that the *per capita* expenses of 2 weeks’ illness, *per capita* hospitalization expenses, annual household income, annual household expenditure, and annual household medical expenses of hypertensive co-diabetic patients were more compared to the patients with the other three comorbidity modes. Most previous studies have looked at diabetes and high blood pressure separately (41–43), not in combination. The results of this study showed that hypertension co-diabetes has brought severe economic burdens. We should not only pay attention to the respective diagnosis, management, treatment, and economic burden of hypertension and diabetes, but also pay close attention to a series of studies on hypertension co-diabetes. Chronic diseases usually do not appear in isolation. Most patients with chronic diseases have multiple diseases (44). Different chronic diseases may influence the development of each other, and the control and treatment of one disease may adversely affect another. Therefore, the management of multiple chronic diseases is a complex public health problem.

### Strengths and limitations

This research is meaningful in health promotion, for the following reasons. This study can provide a broader view of MCCs and its associated factors, by including a larger population, adjusting personal responses, and analyzing related social demographic factors. Firstly, this is a large population-based study on the economic burden of MCCs and associated factors. The data is derived from the Sixth (2018) National Health Service Survey in Yunnan Province, a cross-sectional study. According to the unified requirements and deployment of the National Health and Wellness Commission, the survey samples followed the principle of economic and effective sampling, and adopted the method of multistage stratified cluster random sampling. Secondly, due to the nature of the complex data structure of NHSS, the GEE model was used to adjust the relevant personal responses in this study. Thirdly, we analyzed the influence of urban and rural, ethnic, gender and other social demographic factors on MCCs, which helped us to understand the distribution characteristics of MCCs and may bring policy makers closer to narrowing the gap of observed health inequality. Finally, this study combined diabetes and hypertension to investigate their prevalence and economic burden, which can provide a basis for further research on MCCs, and encourage policymakers and implementers to shift from managing a single chronic disease to the co-management of MCCs.

Our study also has some limitations. Firstly, this study is a cross-sectional analysis, therefore, cannot address a causal (bidirectional) relationship between determinants and MCCs. Secondly, we only analyzed the NHSS data of Yunnan Province, which may limit the generalization of the observed influencing factors of MCCs and their economic burden on a wider population. Additionally, we use the generalized estimation equations model (including behavioral factors, social and economic status factors, health factors, etc.) to control the influence of the confounding factors. However, despite many variables, confounding factors cannot be completely avoided. Finally, there is no standard method to measure MCCs.

## Conclusion

The prevalence of MCCs was relatively high among middle-aged and older individuals in Yunnan, China, which bought a heavy economic burden. This encourages policy makers and health providers to pay more attention to the behavioral/lifestyle factors, that contribute to multimorbidity. Health promotion and education in terms of MCCs must be prioritized in Yunnan. Policymakers and project implementers have only reduced barriers to access to health resources for people with chronic conditions. However, there is a lack of policy orientation for patients with multiple chronic co-morbidities, and accurate identification of the characteristics of multiple co-morbidities is unclear, which in turn affects the ability of the policy to protect the health of the population. At the same time, it cannot reduce the economic burden of chronic co-morbidities in a targeted manner. Therefore, Policy makers in Yunnan urgently need to improve the accuracy of chronic disease co-morbid plan. Adhering to the principle of mutual influence between chronic diseases, while improving the accessibility of medical services for patients with chronic diseases, the next step should focus on improving the chronic disease co-morbid system, to maximize the economic protection for patients with chronic disease co-morbid, to reduce their risk of poverty, and to promote their physical and mental health.

## Data availability statement

The raw data supporting the conclusions of this article will be made available by the authors, without undue reservation.

## Ethics statement

The studies involving human participants were reviewed and approved by the Ethics Review Committee of Kunming Medical University. The patients/participants provided their written informed consent to participate in this study.

## Author contributions

ST designed the study and critically revised the manuscript. PP, JL, LW, ZA, and CT carried out the data export and cleanup work. PP and JL performed data analysis and prepared the draft manuscript. All authors contributed to the article and approved the submitted version.

## Funding

The Department of Education of Yunnan Province’s Funding for the innovation team in universities (K1322114).

## Conflict of interest

The authors declare that the research was conducted in the absence of any commercial or financial relationships that could be construed as a potential conflict of interest.

## Publisher’s note

All claims expressed in this article are solely those of the authors and do not necessarily represent those of their affiliated organizations, or those of the publisher, the editors and the reviewers. Any product that may be evaluated in this article, or claim that may be made by its manufacturer, is not guaranteed or endorsed by the publisher.

## Abbreviations

MCCs: multiple chronic conditions
NHSS: National Health Service Study
GEE: generalized estimating equations
BMI: body mass index
VAS: visual analog scale
OR: odds ratio
CI: confidence interval
